# Carotenoid Derivates in Achiote (*Bixa orellana*) Seeds: Synthesis and Health Promoting Properties

**DOI:** 10.3389/fpls.2016.01406

**Published:** 2016-09-21

**Authors:** Renata Rivera-Madrid, Margarita Aguilar-Espinosa, Yair Cárdenas-Conejo, Luz E. Garza-Caligaris

**Affiliations:** ^1^Unidad de Bioquimica y Biologia Molecular de Plantas, Centro de Investigación Científica de Yucatán A.C.Mérida, Mexico; ^2^CONACYT Laboratorio de Bioingeniería, Universidad de ColimaCoquimatlán, Mexico

**Keywords:** achiote, annatto, apocarotenoids, bixin biosynthesis, anti-cancer, antigenotoxic, antioxidant, hypoglycemic

## Abstract

*Bixa orellana* (family Bixaceae) is a neotropical fast growing perennial tree of great agro-industrial value because its seeds have a high carotenoid content, mainly bixin. It has been used since pre-colonial times as a culinary colorant and spice, and for healing purposes. It is currently used as a natural pigment in the food, in pharmaceutical, and cosmetic industries, and it is commercially known as annatto. Recently, several studies have addressed the biological and medical properties of this natural pigment, both as potential source of new drugs or because its ingestion as a condiment or diet supplement may protect against several diseases. The most documented properties are anti-oxidative; but its anti-cancer, hypoglucemic, antibiotic and anti-inflammatory properties are also being studied. Bixin’s pathway elucidation and its regulation mechanisms are critical to improve the produce of this important carotenoid. Even though the bixin pathway has been established, the regulation of the genes involved in bixin production remains largely unknown. Our laboratory recently published *B. orellana’s* transcriptome and we have identified most of its MEP (methyl-D-erythritol 4-phosphate) and carotenoid pathway genes. Annatto is a potential source of new drugs and can be a valuable nutraceutical supplement. However, its nutritional and healing properties require further study.

## Introduction

**FIGURE 1:**
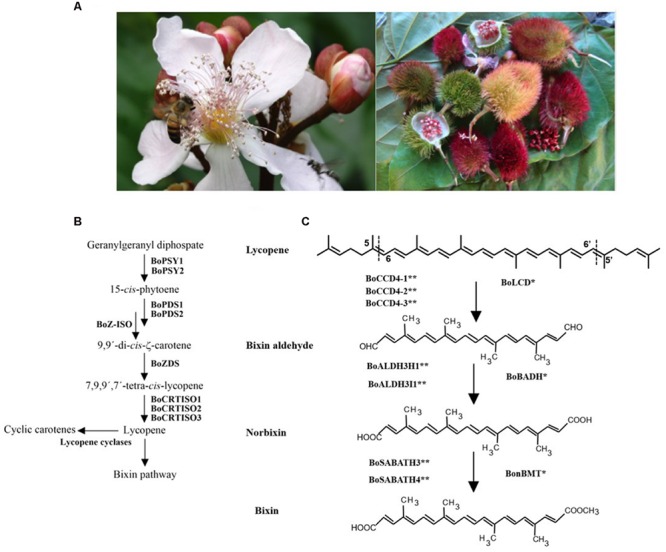
**(A)** *Bixa orellana* L. buds, flower (left), fruit and seeds (right). **(B)** Carotenoid biosynthesis Pathway. Carotene biosynthesis start with the phytoene synthesis by condensation of two geranilygeranyl diphosphate molecules mediated by phytoene synthase enzymes (BoPSY1 and BoPSY2). The phytoene is convert to Lycopene by successive steps of desaturation (phytoene desaturase: BoPDS1 and BoPDS2; ζ-carotene desaturase: BoZDS) and isomerization (ζ-carotene isomerase: BoZ-ISO; Carotenoid isomerase: BoCRTISO1, BoCRTISO2 and BoCRTISO3). **(C)** Bixin biosynthesis pathway. Lycopene is convert into bixin by three serial step reactions. (1) Lycopene is cleaved at the 5-6 and 5′-6′ double bonds by carotenoid cleavage enzymes from family 4 (BoLCD; BoCCD4-1: KT359022; BoCCD4-2: KT359023; BoCCD4-3: KT359024). (2) Bixin aldehyde is the oxidation product of aldehyde groups by aldehyde dehydrogenase enzymes (BoBADH; BoALDH3I1: KT359036; BoALDH3H1: KT359033). (3) Norbixin is converted into bixin by the addition a methyl group in carboxyl groups by a methyl transferase enzyme belonging to SABATH methyl transferase family (BonBMT; BoSABATH3: KT359051; BoSABATH4: KT359052). Dashed lines indicate the position of lycopene cleavage. A single asterisk indicates the proved enzymes that convert lycopene into bixin. A double asterisk indicates the proposed new set of genes involved in bixin synthesis.

*Bixa orellana* L. (family Bixaceae) is a neotropical species, commonly known as achiote in Mexico. *Bixa orellana* was probably domesticated from *Bixa urucurana* Wild (Ambrósio Moreira et al., 2015). This perennial, rapidly growing tree is of great agroindustrial interest because of its seeds have a high carotenoid content, mainly bixin (Rivera-Madrid et al., 2006), (**Figure 1A**). The natural achiote pigments are commercially known as annatto (E160b), and their main orange-red colored component is bixin. It has been used for many years as a dye in various food products such as dairy and bakery products, vegetable oils, beverages and dietary supplement (Dendy, 1966; Tirimanna, 1981). It is also used in the textile, paintings, and cosmetics industries mostly (mainly suntan lotions), (Giuliano et al., 2003).

Bixin was the first *cis*-carotenoid to be isolated from natural sources, (Mercadante et al., 1996); some seeds contain bixin at levels as high as 80 per cent of the total pigment. However, a wide variety of apocarotenoids, including linear and cyclic molecules, can also be found (Mercadante et al., 1996; Bittencourt et al., 2005).

Several studies have addressed the biological and medical properties of this natural pigment. This oil-soluble carotenoid lacks pro-vitamin A activity (De-Olivera et al., 2003), and is one of the more effective biological singlet molecular-oxygen (^1^O_2_) quenchers and scavengers of free radicals (Kovary et al., 2001). Several research groups have also been studying *Bixa orellana* anticancer and apoptotic properties (Coronado-Cáceres et al., 2014). These nutritional and healing properties require further study.

## Characteristics and Properties of Bixin

Bixin is a lineal apocarotenoid of 25 carbon atoms with 9 double bonds and a molecular weight of 394.5 g/mole (Francis, 1987; Britton et al., 2004); its molecular empiric formula is C_25_H_30_O_4,_ and its scientific name is methyl hydrogen 9′-*cis*-6,6′-diapocaroteno-6,6′-dioate ester (Preston and Rickard, 1980; Mercadante et al., 1996). Apocarotenoids are terpenoid compounds derived from the oxidative cleavage of carotenoids (Auldridge et al., 2006; Walter et al., 2010). Seed extracts contain a wide variety of apocarotenoids, including both linear (i.e., methyl (9 Z)-apo-8′-lycopenoate) and cyclic molecules (all-E)-8′-apo-β-caroten-8′-oate) (Mercadante et al., 1996, 1997). Lycopene is described as bixin precursor (**Figure 1B**), although it may also have other non-studied biosynthetic pathways, since bixin is produced despite the inhibition of carotenogenesis using the highest concentration of norfluorazon, a phytoene desaturase inhibitor (Rivera-Madrid et al., 2013).

Bixin has two different stereochemical configurations: *cis*-bixin and *trans*-bixin. The former *cis* is soluble in most polar organic solvents to which it imparts an orange color and is largely insoluble in vegetable oil (Mckeown and Mark, 1962; Francis, 1987; Scotter, 1995; Scotter et al., 2002). It may be readily converted to the all-*trans*-isomers due to the instability of the isolated form in solution. *Trans*-bixin is a more stable isomer, it exhibits a red color in solution and is soluble in vegetable oil (Francis, 1987; Scotter, 2011).

Commercially, isomerization is achieved by heating a suspension of the *cis*-isomer in oil to 130°C *in vacuo*. The water-soluble analog 9′-*cis*-norbixin can be isolated from annatto seeds by agitation in aqueous alkali at <70°C or formed by alkaline hydrolysis of *cis*-bixin to obtain either the sodium or potassium salt (Carvalho, 1992; Scotter, 2011). Bixin is sensible to light, temperature, air, anti-oxidants, and pro-oxidants, and pH (Najar et al., 1988). Its isomers have a maximum absorption with 500 and 470 nm using chloroform (Scotter, 2009). Its fusion point is 189.5–198.5°C (dos Santos, 2007).

Toxicological data on annatto pigments are limited, possibly because food additives derived from natural sources have been exempt from certification (Hallagan et al., 1995). The Joint FAO/WHO Expert Committee on Food Additives (JECFA) estimated the ADI (Acceptable Daily Intake) for annatto as 0–2.5 mg/Kg body weight day^-1^ expressed as the pure pigment (JECFA, 2012). Annatto’s use is permitted for use in food commodities such as savory snack products, coated nuts, extruded products and flavored breakfast cereals. Although JECFA does not allow its use in spices (Scotter, 2011), it is extensively used as such in Mexico, Central and South America. The amounts of the active pigments, bixin and norbixin in annatto can vary from less than 1% to over 85%, depending on the type of annatto extract (e.g., water, vegetable oil or, solvent) (Tennant and O’Callaghan, 2005; Scotter, 2011), and depending on the seed source, as bixin concentration differs greatly among plant variants (Rivera-Madrid et al., 2006).

## Bixin Biosynthesis in *Bixa orellana*

Plant carotenoids have a crucial role in photosynthesis helping to collect light and conferring protection against its excess. Carotenoids are also important precursors of bioactive compounds, such as apocarotenoids which are important in several physiological processes, such as retinol in humans and abscisic acid in plants. Most apocarotenoids are carotenoid degradation products bio-catalyzed by carotenoid cleavage oxygenase enzymes (CCDs). Similar to others apocarotenoids pathways, the biosynthesis pathway of bixin, elucidated in the early 2000 s, involves carotenoid cleavage by CCDs enzymes; the first step is lycopene cleavage in 5–6 and 5′–6′ double bonds (**Figures 1B,C**).

Based on expressed sequences tags (ESTs) library from immature seeds, the first bixin biosynthesis pathway was proposed by Jako et al. (2002). They found cluster of genes related to dioxygenase, aldehyde dehydrogenase and methyl transferase genes with high number of ESTs, suggesting that bixin pathway should be similar to abscisic acid pathway and that bixin’s precursor is a C_40_ carotenoid, probably lycopene, which is converted to bixin by dioxygenase, aldehyde dehydrogenase and methyl transferase genes (Jako et al., 2002). Additionally, they found cluster of genes expressed in immature seeds, where the main production of bixin takes place, related to 1-Deoxy-D-xylulose-5-phosphate synthase (DXS), 1-Deoxy-D-xylulose-5-phosphate reductoisomerase (DXR), 4-Hydroxy-3-methylbut-2-en-1-yl diphosphate synthase (HDS) and 4-Hydroxy-3-methylbut-2-enyl diphosphate reductase (HDR) from methyl-D-erythritol 4-phosphate (MEP) pathway and Phytoene synthase (PSY), Phytoene desaturase (PDS) and ζ-carotene desaturase (ZDS) from carotenoid pathway.

Simultaneously, Bouvier et al. (2003) proposed a similar bixin pathway; they hypothesized that bixin pathway should be similar to saffron pigment crocetin and that the reaction could implicate a dioxygenase, an aldehyde dehydrogenase, and a methyltransferase enzyme that converted lycopene to bixin in serial step reactions (Bouvier et al., 2003) (**Figure 1C**). They identified and isolated a family 4 dioxygenase (*BoLCD*), aldehyde dehydrogenase (*BoBADH*), and methyltransferase (*BonBMT*) genes. These genes were introduced into engineered *Escherichi coli* lycopene producer; transformed bacterias were able to convert lycopene to bixin (Bouvier et al., 2003).

Although bixin pathway has been established, the expression regulation of genes involved in bixin production is unknown, perhaps because the MEP and carotenoids pathways genes, as well as the transcription factor that regulate them remain unaddressed. Recently, during the *B. orellana* transcriptome analysis, the authors identified most of its MEP and carotenoid pathway genes for this plant (Cárdenas-Conejo et al., 2015). Interestingly, a quantitative real time PCR (qRT-PCR) showed that *BoDXS2a, BoPDS1* and *BoZDS* genes were overexpressed in immature seeds, where most bixin is produced, as compared to leaves, whereas carotenoids pathway genes downstream of lycopene were not overexpressed (Cárdenas-Conejo et al., 2015).

Surprisingly, the three genes identified by Bouvier et al. (2003) were not present in *B. orellana* transcriptome, and may have been misplaced in the original study (Cárdenas-Conejo et al., 2015). Based on subcellular localization prediction, function of homologous proteins and qRT-PCR quantification, Cárdenas-Conejo et al. (2015) proposed a new set of genes involved in the conversion of lycopene into bixin (**Figure 1C**); enzymatic activities for this new set of genes need to be characterized.

The enzymes involved in bixin production are present in most plants. Since these enzymes play other important metabolic roles, finding other plants with the ability to produce bixin is not surprising. *Crocus sativus, Vitis vinifera*, and *Costus pictus* produce bixin in detectable levels (Siva et al., 2010; Annadurai et al., 2012). The high quantity of bixin produced in *B. orellana* immature seeds is likely due to the gene expression synchronization of the expression of the genes involved in bixin production, including MEP and carotenoid pathways genes.

Cárdenas-Conejo et al. (2015), proposed an hypothetical model for bixin production in *B. orellana* immature seeds involving the coordinated expression of MEP, carotenoid and bixin pathway genes: (1) MEP genes involved in generation of carotenoids precursors, such as BoDXS2a, BoDXR and BoHDR are induced to produce carotenoids in non-photosynthetic tissue. Enzymes from the DXS2 clade, but not from the DXS1 or DXS3 clades, are involved in carotenoid and apocarotenoid accumulation in non-photosynthetic tissues (Floss et al., 2008; Peng et al., 2013; Saladié et al., 2014). (2) Similar to the tomato ripping process, lycopene cyclase genes from *B. orellana* are turned off, thus blocking metabolic flow toward cyclic carotenoids down-stream of lycopene. The low concentrations of cyclic carotenoids induce the expression of *BoPDS1* and *BoZDS* and promote lycopene production. (3) The bixin pathway genes are then turned on, leading to the conversion of lycopene into bixin.

Full elucidation of the molecular mechanisms that govern bixin production will help understand the mechanisms responsible for the variation of bixin accumulation in *B. orellana* varieties and identify the candidate genes for genetic improvement of this plant to enhance the bixin production.

## Promising Applications of *Bixa orellana* in Medicine

*Bixa orellana* has been extensively used since pre-hispanic times in America as a remedy for different illness. Now a days achiote trees are still used in many communities as a source of treatment for many diseases. During the XVII and XVIII Centuries, it spread widely to countries in Asia and Africa, where it also became part of the ethnobotanical cultural heritage. Although many of these properties have not been studied by modern science, the similarities of the uses given by different cultures could credit certain effectiveness, and shows the relevance of the studying its active principles. Ethnobotanical researchers have documented the use of different parts of the plant, especially leaves, seeds and roots (Kumar et al., 2009; Dike et al., 2012). In Southern Mexico it is used for smallpox and other rashes. It is also used in digestive illness, such as diarrhea, abdominal pain, indigestion and dysentery. Other uses include headaches and sore throat, as abortive, to cure urinary illness and against gonorrhea, and as an abortive agent (Ini-Unam, 2009). In Nigeria it is used against malaria, as an antiseptic and anti-bacterial agent, and against rheumatism (Dike et al., 2012). Similar effects have been documented in Brazil, Peru, Colombia, and other countries in Central America (Vilar Dde et al., 2014) and India (Kumar et al., 2009). Modern science has studied just a few of the healing properties attributed to this species, generally using a mixture of compounds extracted from different parts of the plants.

The most documented effect of bixin in medicine is its antioxidant activity. *In vitro* experiments have shown that seed extracts have a high capacity to scavenge reactive oxygen species (ROS), which correlate to bixin concentration in the extracts (Campos et al., 2011). The possible mechanism for this effect is the electron transfer allowed by the double bonds found in this apocarotenoid or the hydrogen abstraction from carotenoid molecule functioning this way as a chain breaking antioxidant (Junior et al., 2005; dos Santos et al., 2012). The authors concluded that this great scavenging capacity could have clinical applications because bixin is capable of acting as an antioxidant by intercepting free radicals generated by commonly used chemotherapeutic drugs. The protective effect of seed extracts administered to cells and animals treated with cisplatin (*cis*-diamminedichloroplatinum II), a potent antitumoral agent with important side effects, has been documented (Silva et al., 2001; Rios et al., 2009). Important antigenotoxic effects were observed as a reduction of chromosomal aberration, ROS generation, lipid peroxidation, and inhibition of renal glutathione depletion. The effect is observed under a non-toxic dose and is a bixin concentration-dependent manner. Junior et al. (2005) reported not only a protective effect against chemotherapy, but also an anti-mutagenic effect on cells subjected to ultraviolet light. Other research papers also document anti-cancer effects of achiote seeds attributed to compounds such as geranylgeraniol, squalene and beta-sistosterol (Kumar et al., 2009). Tibodeau and Isham (2010) suggest that the anticancer effect could be possible because *cis*-bixin exerts its cytotoxic effects via imposition of cellular ROS mediated, at least in part, by inhibition of the thioredoxin = thioredoxin reductase redox pathway. Another possible use of achiote is in the prevention of diabetic complications due to oxidative stress. Rossoni-Júnior et al. (2012) showed that that supplementation with beta carotene and annatto is able to modulate the production of reactive species in diabetic animals.

Other medical uses of *Bixa orellana* might be re associated to other bioactive metabolites present both in leaves and seeds. A recent study validated the anti-inflammatory effect of this plant. Acute inflammation by injection of histamine in rats was inhibited by oral administration of leaf extracts, comparable to loratadine’s effect. A reduction on vascular permeability was observed as a result of reduced expression of biochemical mediators such as nitrogen oxide and VEGF (vascular endothelium growth factor) (Yong et al., 2013).

In an experiment using tocotrienol extract from achiote to prevent osteoporosis, rats with testosterone deficiencies were treated with achiote seed extract. These rats showed less bone damage as compared to not-treated controls, and an increased expression of bone formation genes (Chin and Ima-Nirwana, 2014).

The antimicrobial activity of ethanol extracts of *Bixa orellana* leaves and seeds was tested *in vitro* on seven common microorganisms: *Staphylococcus aureus, Staphylococcus pyogenes, Salmonella typhi, Escherichi coli, Candida albicans, Bacillus subtilis and Pseudomonas aeruginosa*. Annatto extract inhibited growth of both fungi and bacteria. The effects were slightly lower compared to gentamicin for bacteria and nystatin for fungi (Fleischer et al., 2003). The effect of leaves and root alcoholic extracts on a resistant strain of *N. gonorrhoeae* was also tested *in vitro* showing an important growth inhibition, which was greater with leaf extracts (Cáceres et al., 1995).

Giorgi et al. (2013) documented the repellent efficiency of seed extracts using hexane, ethanol, and ethanol/water as solvents. They found repellence activity against *Aedes aegiptii* mosquito ranging from 22 to 90%, being the hexane extract at high concentrations (113.8 mg/ml) the most effective treatment.

*Bixa orellana* is a potential source of new drugs for a variety of conditions, due to the high concentration of carotenoid derived compounds, and probably because of the presence of other metabolites and peptides, some of them still uncharacterized. More pharmacological studies of this promising plant are needed before it can be used in modern medicine.

## Dietary Contribution

Achiote was used as a coloring agent in pre-hisipanc Mayan religious ceremonies and has been used since to color and flavor certain traditional dishes. In Yucatán, Mexico, the pigment is widely used in its internationally recognized local gastronomy. Achiote seeds contribute to human diet in Mexico and other American countries, and achiote pigments are distributed worldwide. Little is known about its protein and peptidic content, and still less about the biological functions of these molecules which could make the consumption of this natural product even more attractive (Coronado-Cáceres et al., 2014). A potential value of this product is the antioxidative function that could reduce the damage caused by free radicals, and be useful in cancer prevention (Reddy et al., 2005). Preliminary studies in our laboratory have given us clues about certain achiote peptides that could be established them as new nutraceutical cancer preventives against cancer (Coronado-Cáceres et al., 2014).

Carotenoids that have antioxidative effects have been identified in achiote; it has also been reported that the ingestion of this condiment reduces triglycerides in plasma (Kiokias and Gordon, 2003). It is well known that a key element in the development of diabetic complications is oxidative stress (Rossoni-Júnior et al., 2012). Levy et al. (1997) found in a study with a group of volunteers that after ingesting a single dose of 1 ml of a commercial annatto food colorant, bixin levels reached high concentrations in human plasma and were completely cleared in 8 h. Thus, the bixin present in processed foods may be an important nutritional factor that can promote human health. A study using carotenoid mixture found that they have antioxidative and anticarcinogenic effects (Reddy et al., 2005). However, more accurate studies need to be developed to demonstrate the specific effect of achiote pigments.

Interestingly, these pigments were found to have hypoglycemic effect using dogs, rats and human volunteers as experimental models (Fernandes et al., 2002; Junior et al., 2005; Russell et al., 2005). Thus annatto extract may have therapeutic potential for diabetes.

## Conclusion

Antioxidant effects of bixin and other achiote compounds have been demonstrated, so its consumption, either as a pigment or spice may provide health benefits. Other properties such as hypoglucemic and anticancer activities are being studied. Preliminary investigations in our laboratory with active peptides from seeds also suggest an effect in cancerous tumors (Coronado-Cáceres et al., 2014). It is important to promote the intake of achiote seeds and pigment in the diet for its medical, nutraceutical and nutritional potential values, as well as to promote its cultivation and production. Elucidation of bixin synthesis in achiote, and metabolite profiles are important research topics contributing to increase produce and use to promote human health. Further studies are still needed before bixin and other achiote compounds can be used extensively by modern medicine.

## Author Contributions

RR-M conceived and designed this manuscript. All authors wrote, critically read, contributed to and commented on the manuscript.

## Conflict of Interest Statement

The authors declare that the research was conducted in the absence of any commercial or financial relationships that could be construed as a potential conflict of interest.
